# Nursing Students’ Experiences in Consecutive Clinical Interprofessional Education in Japan: Application of the IPE in Nursing Colleges

**DOI:** 10.3390/nursrep12020032

**Published:** 2022-04-29

**Authors:** Junko Shida, Mariko Otsuka

**Affiliations:** 1 School of Nursing, Yamagata University, 2-2-2 Iida-Nishi, Yamagata 990-9585, Japan; 2 School of Nursing, Nagano College of Nursing, 1694 Akaho, Komagane 399-4117, Japan; motsuka@nagano-nurs.ac.jp

**Keywords:** clinical practice, interprofessional work, interuniversity collaboration, practice experience, interprofessional education

## Abstract

Aim: To document nursing students’ experiences of continuous participation in a clinical interprofessional education (IPE) program with the Faculty of Pharmacy of other universities in Japan, which had been incorporated into the existing practicum program, and consider how to develop a one-shot clinical IPE program. Methods: The study participants were two nursing students from a single-department college; they were participating in a clinical IPE program—the first IPE program they had ever participated in—during an area-based practicum in Year 3. Subsequently, in Year 4, a semi-structured interview was conducted, and these interview data were qualitatively and inductively analyzed. Results: Seven categories were identified, and it was found that through continuous participation in the IPE program, there was a change from “clinical IPE is stuck at the back of their mind as a bitter experience” to “the process of clinical IPE stimulates their maturity as a nursing student” and “cultivates attitudes necessary for cooperation”. Conclusion: Consecutive years of continuous clinical IPE helps students deepen their understanding of learning content by reducing the physical and mental burden of multidisciplinary cooperation and collaboration. For difficulties with regard to step-by-step IPE, it is suggested that one-shot clinical IPE can be conducted for the upper grades along with continuous reflection activities for teams and individuals.

## 1. Introduction

Health and welfare care professionals must respond to the accelerating diversification and complexity of their subjects’ needs and high healthcare specialization with the aim of providing safe and high-quality care. Currently, considering the critical global shortage of healthcare workers, professionals must provide the highest quality of care in order to improve their subjects’ health outcomes in a challenging environment [1]. This is made possible by Interprofessional collaborative practice (IPC), whereby multiple health workers from different professional backgrounds work together with patients, families, carers, and communities in order to deliver the highest quality of care [1].

It is essential to continuously cultivate the foundational competencies for realizing IPC from the basic education curriculum, and Interprofessional Education; thus, IPE is being promoted worldwide as one strategy for achieving this [1,2,3]. IPE—that is, Interprofessional Education—”occurs when two or more professions learn with, from, and about each other to improve collaboration and the quality of care” [4]. Effective IPE has been found to lead to improved patient outcomes, improved patient safety, and increased staff morale [1]. Thus, IPE is being promoted globally because it has been recognized as an essential step for preparing a “collaborative practice-ready” health workforce that is better prepared for responding to local health needs [1].

A similar trend toward IPE promotion is present in Japanese nursing education. Japan’s nursing education is diverse, with four-year nursing colleges, three-year junior colleges and training schools, and five-year nursing training courses [5]. However, all these curricula hold the acquisition of multidisciplinary collaborative practice skills in a health and welfare team to be the minimum level of competence and content knowledge that all nursing students should achieve upon graduation [6,7]. In order to acquire the ability to work collaboratively, from the point of view of educational effectiveness, it is desirable to teach using a step-by-step IPE involving lectures, exercises, and practical training [8]. However, in Japan, a disparity has been observed in IPE implementation in nursing education, and the rate of this non-implementation is as high as 80%—especially in nursing training schools [9]. The reasons for this situation include the difficulty of coordinating curriculums and timetables with those of other universities or schools [10] and the fact that practice-based IPE implementation in clinical practice, which is expected to have a high educational impact, has not progressed [11,12].

Against the backdrop of the abovementioned issues, University A, which only has a nursing faculty as part of its medical faculty; University B, specifically their School of Pharmacy; and Hospital C initiated “practice-based IPE” (clinical IPE)—a form of practical training where multiple institutions collaborate on the development of the same practice field. While clinical IPE and its unique features were not previously found in Japan, practice-based IPE was incorporated into the existing practice at both universities in 2017 as part of a trial. The trial results showed that, even though the first IPE was implemented as a clinical IPE in the upper grades, the students who participated in this trial improved their basic IPC skills [13,14]. On the other hand, unable to sublimate their negative feelings (e.g., including feelings of inadequacy) [13], which may have led them to doubt the effectiveness of IPE.

Therefore, we decided to focus on experience—which is a subjective awareness process—of nursing students who participated in two consecutive clinical IPEs; these were implemented during the third and fourth years. The subjects included students who experienced negative feelings during the aforementioned clinical IPE [13]. Past IPE practices have been analyzed both qualitatively and quantitatively [15,16,17]. Interaction with other professionals can improve professionals’ understanding and communication skills and also benefit the patients receiving care [14,15]. On the other hand, IPE’s negative effects have been qualitatively identified—mainly, with a focus on student anxiety prior to IPE [16]. Furthermore, focus group interviews have revealed the challenges that students face during IPE; these include differences in knowledge, issues related to equality, and the tendency to interact more with other professionals within the same profession [17]. However, few studies have focused on the overall IPE experience, including individuals’ negative feelings, and these aspects have not been clarified over time. Therefore, we considered that there could be some challenges with regard to reflecting IPE experiences from students’ perspectives—especially with regard to educational content and methods.

Based on the abovementioned considerations, the present study aims to clarify the IPE experiences of a rare group of students, which had a first IPE that was clinical and participated in two consecutive clinical IPEs over time. We considered that this would provide information about their IPE experiences that could not be captured by objective measures; this, in turn, could lead to educational method-related proposals that would take attitudinal readiness into account.

This research thus aimed to document the experiences of nursing students who continuously participated in a clinical interprofessional education (IPE) program (stretching over consecutive years) that had been incorporated into an existing practicum program with Faculties of Pharmacy in other universities in Japan; furthermore, it also aimed to consider ways to develop a one-shot clinical IPE and examine ways to develop an IPE that could be applied to a single university.

## 2. Materials and Methods

### 2.1. Research Design

We used a qualitative descriptive research design in order to uncover the subjects’ experiences. Our study defined “experience” as the facts and actions that occurred in the clinical IPE and the mental movements and awareness that occurred during the process.

### 2.2. Participants

The study target population included seven fourth-year students from the Faculty of Nursing at University A, who had continuously participated in the clinical IPE during their third year (September–November 2017) and fourth year (May 2018). Prior to the third-year discipline-specific practice, the subjects’ field practice experience had included basic nursing practice in the first and second years (approximately one month in total).

Table 1 shows the clinical IPE objectives and development methods. Because of the absence of IPE-specific lectures or exercises at both universities, the participant students were prepared for the clinical IPE through orientation and pre-assignments that were common to the two universities (STEP 1). Faculty members from the School of Nursing and the School of Pharmacy as well as clinical supervisors from the hospitals participated as facilitators and observers in the discussions held during the clinical IPE (STEP 3). Faculty members involved themselves in the discussions if the students did not get stuck or deviate too far from the discussion themes.

### 2.3. Data Collection Methods

During the third- and fourth-year practical orientation, we distributed cooperation request forms to the subjects and requested their cooperation in the study both verbally and in writing. We explained the study again to the subjects on the day of the interview and obtained their consent for participation in the study; the participants’ signing of the consent form was considered as consent.

Following the two clinical IPEs, a face-to-face semi-structured interview was conducted in December 2018. In these interviews, we utilized the interview guide provided in Table 2; questions regarding “the most memorable scene” and “ the situation” in the clinical IPE were utilized in order to enquire about “the most memorable scene” of the clinical IPEs and about the “actions during the clinical IPE,” “feelings,” and “thoughts” as well as “what the situation was.” The content was recorded on an IC recorder with the subject’s consent.

### 2.4. Data Analysis Methods

The audio data from the interviews were transcribed verbatim and the verbatim transcripts were read through to extract what was said about experiences in clinical IPE. The semantic content was separated into the smallest units that could be grasped and coded based on the semantic content. In this process, third-year experiences, fourth-year experiences and common experiences were identified. The codes were then compared based on similarities and differences to form a typology, which was then abstracted into subcategories. The subcategories were repeatedly compared, modified, and refined into categories by content. These series of analyses were conducted with collaborators and in multiples to ensure the truthfulness of the analysis.

### 2.5. Ethical Considerations

This study was approved by the Ethical Review Committee of the affiliated university (Approval No. 161). The researcher provided written and oral explanations to the subjects about the study’s purpose and methods, the voluntary nature of the research cooperation, the data storage, the protection of personal information, and the publication of research results before conducting the clinical IPE and the interview survey.

The students themselves submitted their responses to the questionnaire after the practical training and deposited these directly in a locked box in the university.

To ensure that the presence or absence of research cooperation would not affect evaluation of the students’ performances, cooperation intentions were ascertained after finalizing the practical training. The students were also informed, in advance, that they could withdraw their consent from the research at any time. A teacher who was not overseeing the students’ practical training conducted the survey.

The above-mentioned measures were implemented in order to protect the vulnerable subjects as well as the voluntary nature of the research because teachers were conducting the research with vulnerable students.

## 3. Results

### 3.1. Outline of Participant Information

Two students agreed to participate in the study, and they were both assigned to a practice hospital that incorporated clinical IPE. The interviews lasted from 42 to 51 min per subject (average: 46.5 min).

### 3.2. Overview of the Participants’ Experiences

We extracted 7 categories representing the participant’s experiences in the clinical IPE, 17 subcategories representing the specific experience content, and 106 codes. They were represented as follows: subcategories by <>, narratives in italics, and supplementary interpretations of the narrative content by () when enclosed in quotations.

#### 3.2.1. Entering the Upper Grades without Fully Understanding the Expertise Other Professions

This category comprises two subcategories which indicate that, due to the characteristics of a single college of nursing, students have limited opportunities to interact with pharmacy students and other students who wish to enter other healthcare professions and that they have progressed to higher education without fully understanding the expertise required for practicing other professions.

<Limited access to pharmacy students and pharmacists> revealed that the students had limited opportunities to come into contact with pharmacy students and pharmacists.

*“In our daily lives, the only opportunity to come into contact with pharmacists is when we go to the hospital.”*

*(Student A)*

*“I have very few opportunities to engage with pharmacy students at university.”*

*(Student B)*

Furthermore, the lack of contact with other professionals was linked to a poor understanding of professionalism, as <Not truly understood the specialty of pharmacy>.

*“I didn’t know much about the content of hospital practice (conducted by pharmacy students) or the work of pharmacists, and I didn’t understand it (until receiving IPE in the third year)”.*

*(Student B)*

*“I wondered how ‘How do pharmacy students and pharmacists relate to patients?’ I did not gain much of an understanding of this until receiving this IPE”.*

*(Student A)*

#### 3.2.2. Fostering Attitudes toward Clinical IPE through the Encouragement of Teachers

This category included two subcategories, which indicated the desired state of readiness for the clinical IPE, as explained and encouraged by the teacher.

<The teachers’ explanations and support in visualizing the clinical IPE> showed that teachers’ explanations, prior efforts, and so on showed that students were able to gain perspective on their first IPE.

*“I was able to gain a clear idea of what we would be doing thanks to the orientation.”*

*(Student A)*

*“There are parts that I won’t know until I try it, but I have some idea of what IPE is like.”*

*(Student B)*

In addition, <Encouragement from teachers helped ease tensions and allowed engagement with pharmacy students in a relaxed manner> indicated that the faculty’s encouragement throughout the clinical IPE process helped ease the minds of students and helped them participate in the IPE without becoming overly nervous.

*“The teacher told me that everyone has more experience in practical training than the pharmacy students, so the nursing students are more used to practical training. So, I think I was able to participate without too much fuss.”*

*(Student A)*

*“Before the discussion, the teacher told me that I could and should ask these things to pharmacy students. That made me feel a bit more relaxed and relieved that I didn’t have to grow taller.”*

*(Student B)*

#### 3.2.3. Engaged Directly with Students with Different Specialisms for the Subject in Practical Training

This category included three subcategories representing the aspect of face-to-face interaction with pharmacy students on behalf of the target patients.

<Sharing time and space with pharmacy students and repeatedly engaging with them onsite for the benefit of the subject> allowed students to understand the relationship between nursing and pharmacy students.

*“(About clinical IPE in Year 3) first of all, it was refreshing to be involved with students other than nursing students in practical training… In (clinical IPE) Year 4, we talked to different people, but it was the second time that I talked to pharmacy students in practical training, so the discussion was smooth, and I was able to give more information and comments about the patients I was taking care of. It’s become a lot easier now.”*

*(Student A)*

*“After the first one, the second time, we had more to say to each other compared to during in the third year, so it was a proper catch-up conversation”.*

*(Student B)*

<Engaged with pharmacy students through trial and error, trying to accurately convey the subject’s situation from a nursing perspective> indicated that nursing students struggled to engage with pharmacy students during the direct engagement. The students struggled to communicate certain terms that would be understood without difficulty by other nursing students of the same specialty, but they tried their best to convey and share information about the subject while searching for words that would be understood by the other person.

*“I was surprised or puzzled that pharmacy students couldn’t understand the language that we usually use… So, I made a conscious effort to speak in a way that people who didn’t understand the terms I used could understand them and tried to communicate well and properly about the patients. It was difficult—especially at the beginning.”*

*(Student B)*

*“I was conscious that I was speaking in a way that people who did not understand the terms I usually use would understand, and I was also conscious of communicating well and properly about the patients.”*

*(Student A)*

<In-depth and developmental discussions with pharmacy students to find better care for the subject> indicated that these trial-and-error experiences were not just some bitter experiences and could be used for the second clinical IPE.

*“We discussed the image and goals we wanted to achieve for the patients we receive, sharing information about their lives, their illnesses, their thoughts, and so on… I think the 4th year clinical IPEs were more convincing to each other than in the 3rd year, and we derived goals for the patients.”*

*(Student A)*

*“(IPE) in year 4 was the second time, and I think it was partly because I was used to it, but it was interesting because we were able to discuss more in depth and what kind of care would be best for that patient. If I had only talked with nursing students, as I have done in the past, I think I would have only been able to provide more narrow-minded goals for the patient. So I feel fulfilled.”*

*(Student B)*

#### 3.2.4. Clinical IPE Is a Bitter Experience That Sticks with You

This category represented the negative feelings that welled up during the clinical IPE and continued to linger in the students’ minds; it consisted of two sub-categories.

The category <Negative feelings about the previous clinical IPE have not disappeared and are still smoldering> presented an added burden in that the students had to do their first IPE while they had not had time to mentally prepare for the start of their domain-specific training. Such negative feelings were also indicated by the fact that the participants had not found any results or had experienced unrefreshing feelings during the clinical IPE discussion.

*“I really didn’t like the added burden [in the area-based training], even though I was full of myself.”*

*(Student B)*

*“Nursing students and pharmacy students were so reserved with each other that we didn’t get to talk much.”*

*(Student B)*

*“I ended up halfway through because I had unresolved questions and did not know what I should have done.”*

*(Student A)*

In the clinical IPE year 4, the students experienced these negative feelings that had not been sublimated, thus leading to their reluctance to participate in IPE again, as indicated by <Not feeling comfortable with the new IPE because of the sense of inadequacy with the previous clinical IPE >.

*“I thought I would be made to participate [in IPE] again. It was daunting.”*

*(Student B)*

*“I wasn’t looking forward to it or actively wanting to take part [in IPE]. If anything, I didn’t feel like it.”*

*(Student A)*

#### 3.2.5. Ensure That the Nursing Profession and Pharmacists Are Integral to Each Other

This category included three subcategories, which represented the aspect that, through learning collaboratively with pharmacy students, they became clear and realized that both sides are close and irreplaceable colleagues who support healthcare.

<Experiencing firsthand the depth of the pharmacy profession through my interactions with pharmacy students> expressed a sense of respect for the high level of expertise required for practicing pharmacy and the attitude that pharmacists are an essential part of the health and social care team.

*“We look into drugs too, but pharmacy students are still even more amazing than that.”*

*(Student B)*

*“They really taught us things we didn’t know, like this medicine can be stopped”.*

*(Student A)*

<In interactions with pharmacy students, sensed that they respected us as a person and recognized the importance of nursing> indicated that the nursing students expressed pleasure that, when they were exposed to the polite attitudes, language, and behavior of the pharmacy students, they perceived that they were respected as human beings and that nursing was valued by other professionals.

*“I asked a question about something I didn’t understand, and they looked it up and told us before the next time, which I appreciated.”*

*(Student A)*

*“It is best to ask the nurses about the patients, as they are the ones who are closest to the changes, and I was glad that [pharmacy students] saw and appreciated nursing in that way.”*

*(Student B)*

In addition, <A sense that nurses and pharmacists are equal colleagues in supporting healthcare> indicated that they realized that both sets of students were aiming in the same direction and that they were equal colleagues who form a team with the subject at the center of the team.

*“The nurses wanted to support the patients in realizing their wishes, and the pharmacists felt the same way. (I realized that they intervene with medicines [for this purpose].”*

*(Student B)*

*“We’re from different grades and specializations, but I enjoyed talking to them as an equal more than I thought I would.”*

*(Student B)*

*“Pharmacists have different specializations compared to nurses, but there are commonalities and overlaps. I was convinced that we have the same goal of helping patients and making them better.”*

*(Student A)*

#### 3.2.6. Maturity as a Nursing Student Is Encouraged through the Clinical IPE Process

This category represents how growth as a nursing student is facilitated through the clinical IPE process; it was organized according to three subcategories.

<Reflecting on clinical IPE and making sense of its value for ourselves> indicated that students reflected on the clinical IPE process and described how they themselves had found meaning in it.

*“There are tough times, but there are also things that I realized because I was involved with pharmacy students… IPE has confirmed for me that knowledge from textbooks is important for performing IPE and that this is what collaboration is all about ”.*

*(Student B)*

*“Because I did the IPE, I could really feel and understand what collaboration is and what this is all about. It became clear to me.”*

*(Student A)*

In addition, <Develop skills to deal with similar situations through clinical IPE> indicated that the participants reflected on their experiences during IPE and showed how they applied them to their practice and life.

*“I started to think about my choice of words, for example, whether it would be better to supplement my explanation with more words here for people outside the nursing profession.”*

*(Student A)*

*“In IPE, I noticed again that I tend to give up explaining things early on if the story seems hard to get across. I didn’t realize that when I was dealing with nursing students because I could get through to them. I now have to be more careful when talking to people with different specializations, and I can be more objective in order to be careful because I seem to get into bad habits.”*

*(Student B)*

Furthermore, the students were shown how <Putting into words what the nursing profession should be and wants to be> and the IPE helped them to reconfirm their own views on nursing reaffirm their nursing expertise.

*“Only nurses can quickly see changes in patients’ bodies and lives over time, and the IPE reaffirmed that this is our strength.”*

*(Student A)*

*“I realized that it is the nurse’s role to inform (other professionals) about aspects of the patient’s life and concerns.”*

*(Student B)*

#### 3.2.7. The Attitudes Needed for Collaboration Are Developed

This category comprised two subcategories, which showed how participation in clinical IPE can shape attitudes and change behaviors to allow students to interact with other professions in an unpretentious way.

<Increased interest in other professions and motivation to work on their own> was described as a positive attitude that drew attention to the movement of multiple professions and encouraged them to work on their own towards other professions.

*“I noticed the presence of pharmacy students, raised my hand and bowed to them as a familiar face, and they smiled and answered me.”*

*(Student A)*

*“I saw the nurses talking to the pharmacist and paid attention to what they were saying.”*

*(Student B)*

*“I asked the pharmacist questions about what he talked about in the drug instruction, what he paid attention to, and what he told the patients.”*

*(Student A)*

Thus, it could be said that these had changed to a state where the barriers, both physical and mental, to interacting with others with different specializations were disappearing, as in <Feel less stressed, both physically and mentally, when interacting with others who have different expertise> (Table 3).

*“Now I don’t feel that it is so much of a burden to cooperate and relate to others.”*

*(Student A)*

*“A pharmacist came to my patient’s place and I sat in. … I am now able to talk to them without being too nervous, even if they are from other professions, like ‘may I join you?”*

*(Student A)*

*“I think there were a lot of barriers before IPE. Like being nervous about what to talk about with people from other professions. Now I don’t feel those barriers so much.”*

*(Student B)*

## 4. Discussion

Even though the IPE that the students experienced for the first time was a limited development method of clinical IPE that was incorporated as a part of their existing practice, the students successfully cultivated the skills necessary for multidisciplinary collaboration through continuous participation in the clinical IPE and reflected generally on the experience; this led to their growth as nursing students. Based on these aspects of the experience, this paper discusses a method of developing IPE that can be applied to a single college.

### 4.1. The Nature of and Factors Underlying the Experience of Students Who Continued to Participate in the Clinical IPE

The first IPE that the students experienced during their senior year was a practice-based IPE with advanced practice. While staged IPE is considered ideal [8], it is a contradictory method, as indicated by previous studies [1,3,15,17]. In this regard, one factor that was suggested as an improvement for the ability to collaborate, including the understanding of expertise (Category 5, 7), was the accumulation of knowledge and skills in nursing. The interviews provided narratives that learning became clearer through the clinical IPE process (Category 6). The older students, who had a broader knowledge base for nursing practice than the younger students, were able to use inductive thinking to organize the various phenomena they experienced during the clinical IPE process with the support of their teachers. The use of deductive thinking also allowed these students to apply theories and other abstract concepts during the clinical IPE.

Thus, it was thought that the clinical IPE experience was linked to their learning up to that point and led to real understanding. The older students also enhanced their communication skills compared to when they first entered the program [18]. Therefore, they were more encouraged to form and develop relationships with pharmacy students, and it could be said that the real-life experience of having clinical collaborative discussions regarding benefits for the patients under their care (Category 3, 5) enhanced the learning effect. These aspects of clinical IPE as experiential practice education [19] conveyed the attractiveness and fun of nursing to the students and, as a result, the processes and learnings of clinical IPE were engraved in the students’ minds.

On the other hand, the students also perceived the clinical IPE they received in the third year as a negative experience (Category 4) and only perceived it to have a positive meaning in the fourth year (Category 6). This situation could be attributed to their ability to objectify the experience away from the practice. It has been pointed out that, during a stressful experience, it is necessary to temporarily distance oneself from the experience because trying to find meaning immediately afterwards can amplify the stress [20]. Furthermore, deliberate contemplation to find the value and importance of the event increases over time, leading to a positive re-evaluation of the event and a positive change in personal beliefs and values [21]. This interview was conducted with a fourth-year student. The fact that this interview took place more than six months after the fourth-year clinical IPE may also have facilitated a positive value shift.

Furthermore, during the clinical IPE in year 4, students developed a stronger sense of community in that they perceived nurses and pharmacists as equal colleagues in supporting healthcare (Category 5). A sense of community refers to a feeling of always being connected to others because of weaknesses, shortcomings, and limitations [22]. Through clinical IPE, students focused on the similarities and differences between nursing and pharmacy and were reminded that nursing is a broad and highly specialized field that advocates for life and living and has various strengths (Category 6). On the other hand, while realizing the overlap in expertise, they also recognized that pharmacy students have knowledge, skills, and experiences regarding pharmacy that set them apart from nursing students (Category 5). Thus, professionals from different fields exist and team up in order to ensure that they complement each other’s expertise for the benefit of the subject. It is thought that these successes, including the deepening of their understanding of professionalism, encouraged the students to confront their bitter experiences and thus led to their positive psychological transformation.

The attitude of considering other professionals as peers is something that is experienced in the early years when IPE is implemented in stages. In this respect, students who participate in IPE at an older age may be disadvantaged, as they are less likely to have real-life understanding. However, IPE is still an important opportunity for students to create their own beliefs and values regarding how cooperation and collaboration can be essential in health and social care by recognizing the commonalities between other professionals and gaining convincing experiences of the importance of multidisciplinary teams through this basic education course. Since beliefs and values may influence behavior more than knowledge [23], even a one-off clinical IPE is considered to be of great significance as an addition to nursing practice.

### 4.2. Investigation of a Single Clinical IPE to Be Incorporated into Existing Nursing Practice

Clinical IPE, which allows direct interaction with students who aspire to practice other professions in an ever-changing and tense clinical environment, is an important learning opportunity that has the potential to further develop certain potential abilities of students, which cannot be strengthened by nursing practice alone. Therefore, for nursing colleges that may experience some difficulties in implementing IPE in stages (considered to be an effective strategy), the current study results suggest a method of implementing one-off clinical IPE for older students based on the development of their learning readiness. In the implementation of IPE, the active use of Information and Communication Technology; ICT (implemented during the meeting stage before the clinical IPE) [24] is also useful, and it is thought that it can overcome several obstacles that single colleges face (for example, the adjustment of the time schedule and practice time and the physical distance between facilities).

The importance of reflection was reiterated through students’ verbalization of their learning and clarification of its meaning as well as the lessons they learned from it. In a one-off clinical IPE, it is important to encourage students to reflect, face-to-face or via ICT, with other professionals who have experienced the IPE together and to strengthen their own reflections. Furthermore, teachers must deliberately ask questions about the clinical IPE in the interviews after the clinical IPE—that is, at the time of integration practice— and at graduation, as this will complement the one-off IPE and provide an opportunity for students to make sense of what they considered to be a negative experience and thus generate new lessons.

Finally, limitation of this study was the possibility that it had a sampling bias. It could also be pointed out that it was a pilot study due to size of target population. Clinical IPE in this study is “a form of practice developed by multiple institutions collaborate in the same practice area”, which is unique in Japan and has been incorporated into practice as a trial. Moreover, the study’s target population was limited by the fact that very few students have a first IPE that is clinical in a clinical setting and have participated in two consecutive clinical IPE sessions. Moreover, the voluntary nature of participation in the current study was guaranteed, thus resulting in a very small number of participants (two). These factors mean that, while the data from this study are of great value, there are limitations to the analogy of the results. Although, it should be noted, due to the above peculiarities, that despite the population size of this study being extremely small, the nature of this study differs from pilot studies that are conducted with fewer participants on a preliminary basis. These limitations of the system of analogy can be worked upon by accumulating more data.

Furthermore, the possibility that a Hawthorne effect [25] may have occurred cannot be ruled out. The clinical IPE in this study was in a trial phase and only a small number of students were able to participate in it. This can be rephrased as an “event of different practice and experience” for the subject. Moreover, university teachers and practice supervisors participated as observers in discussions with pharmacy students, which may have created a sense of being “evaluated” due to the nature of the practice. These situations may have elicited a sense of motivation and responsibility from the students and thus improved competence due to their desire to meet expectations by receiving attention from others. In the present study, these points should be considered when interpreting the results. It should be noted that the interview survey combined with the fact that it was conducted by teachers, may have led to biased results towards positive evaluations.

Moreover, there were limitations in data collection because of the recalling of past experiences. Therefore, it is necessary to consider methods that can enable more vivid recollection, such as utilizing practical training records and video data from clinical IPE during the interviews. Furthermore, as this study did not include the contemplations of the pharmacy students who undertook the practical training together, it is necessary to compensate for the limitations of this study by conducting analyses with pharmacy students in the future.

## 5. Conclusions

The nursing students who continued to participate in the clinical IPE in their third and fourth years, as part of their existing practice, confirmed their awareness of each other as an integral part of healthcare through their interactions with pharmacy students and reflected on their experiences. By reflecting on their experiences, they were encouraged to grow as nursing students, which helped them to develop as nursing professionals. It became apparent that a spontaneous attitude of collaboration was developed.

This study found that, even if there may be some difficulties in conducting IPE in stages during the early grades at a single college, it is important to conduct clinical IPE, even if it is only a one-time event. To increase the effectiveness of clinical IPE, this study suggested that the target group should be older students who have acquired more basic knowledge and skills in nursing, that interaction among students should be promoted by using ICT before clinical IPE, and that team reflection and continuous individual reflections are necessary.

## Figures and Tables

**Table 1:** Objectives and development of clinical IPE.

Objective of the Clinical IPE: To Learn about Cooperation and Collaboration between Health and Social Care Professionals	
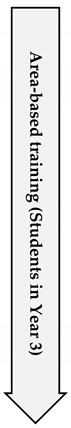	STEP 1: Basic learning to increase readiness: Common to all practical training (conducted at each university) Orientation to practical training Presentation of a pre-assignment common to the two universities: a report on team medicine etc. Lecture on the basics of IPE
	STEP 2: Practical training in the hospital (each student) Interaction with the patient (nursing and pharmacy students participate in the care together) Observation of collaborative situations in clinical practice
	STEP 3: Discussion with nursing and pharmacy students based on STEP1&2 (60~90 min) Icebreaker Sharing of information and common understanding of the patients in your care The theme of discussion Review of patient support goals (60 min × 2 times) Based on the discharge support provided to patients, discuss the assistance and points to be considered in providing discharge support from the perspective of multidisciplinary cooperation (90 min × 1 time)
	STEP 4: Reflection in each university and training area
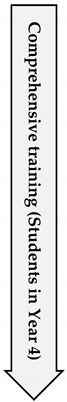	STEP 1: Basic and developmental learning to increase readiness: Common to all practical training (conducted at each university) Orientation to practical training Presentation of a pre-assignment common to the two universities: a report on team medicine etc. Lecture on the basics of IPE
	STEP 2: Practical training in the hospital (each student) Interaction with the patient (nursing and pharmacy students participate in the care together) Observation of collaborative situations in clinical practice
	STEP 3: Discussion with nursing and pharmacy students based on STEP1&2 (60~90 min) Icebreaker Sharing of information and common understanding of the patients in your care The theme of discussion Setting goals and reviewing and presenting support for patients Behaviors and attitudes that are important for professionals to work together
	STEP 4: Reflection in each university and training area

**Table 2:** Interview Guide.

1. What was the most memorable moment during the clinical IPE? 1-1. What was the situation? 1-2. How did you feel and act? 1-3. How did the people around you behave? 1-4. What do you think were the reasons for your impression?
2. What were your impressions of students from other faculties and other professions after doing clinical IPE? 2-1. What was your impression before clinical IPE? 2-2. What are the reasons for this? 2-3. Have these feelings changed since completing the clinical IPE? 2-4. If it has changed, please explain why? 2-5. Were there any new findings?
3. Did you learn from the clinical IPE? 3-1. What learnings have you gained? 3-2. Do you think you can make use of this experience in the future? 3-3. How do you think you can make use of this experience?

Used as a reference in encouraging narrative rather than for all content.

**Table 3:** Experience in clinical IPE.

Category	Subcategories	Example of a Code (Time of Experience)
1. Entering the upper grades without fully understanding the expertise of other professions	Limited access to pharmacy students and pharmacists	In my daily life, the only time I come into contact with a pharmacist is when I go to the hospital (Year 3)
	Not truly understood the specialty of pharmacy	I didn’t know what pharmacy students practice and they do until my third year (Year 3)
2. Fostering Attitudes toward clinical IPE through the encouragement of teacher	The teachers’ explanations and support in visualizing the clinical IPE	There was also an orientation, which gave me some idea of what I would be doing (Year 3 & 4).
	Encouragement from teachers helped ease tensions and allowed engagement with pharmacy students in a relaxed manner	The teacher’s encouragement made it easy for me to work without being so overwhelmed (Year 3 & 4).
3. Engaged directly with students with different specialisms for the subject in practical training	Sharing time and space with pharmacy students and repeatedly engaging with them onsite for the benefit of the subject	We were saying more to each other than we did in Year 3 and were catching up properly (Year 4).
	Engaged with pharmacy students through trial and error, trying to accurately convey the subject’s situation from a nursing perspective	It was difficult to try to explain in words because they kept asking us what we meant by the terms we normally used (Year 3)
	In-depth and developmental discussions with pharmacy students to find better care for the subject	The fourth-year students were able to agree with each other and come up with more goals for their patients than the third-year students (Year 4)
4. Clinical IPE is a bitter experience that sticks with you	Negative feelings about the previous clinical IPE have not disappeared and are still smoldering	I didn’t want to be nervous because I was so busy with my own work in the different areas, but it added another burden to my work (Year 3) There were questions and things I couldn’t digest, and I ended up feeling half-hearted (Year 3)
	Not feeling comfortable with the new IPE because of the sense of inadequacy with the previous clinical IPE	I wasn’t keen on doing clinical IPE again before I did it (Year 4)
5. Ensure that the nursing profession and pharmacists are integral to each other	Experiencing firsthand the depth of the pharmacy profession through my interactions with pharmacy students	It is totally different from the amount of knowledge we have about drugs (Year 3 & 4) Really taught me what I didn’t know about medicine (Year 3 & 4).
	In interactions with pharmacy students, sensed that they respected us as a person and recognized the importance of nursing	I appreciated the fact that if I didn’t understand something, they would look it up and teach me before the next time (Year 3 & 4) I was happy to see that pharmacy students appreciated nurses as they were the best people to ask about patients and the ones who were closest to see changes (Year 3 & 4)
	A sense that nurses and pharmacists are equal colleagues in supporting healthcare	Pharmacists also work together with the same goal in mind, which is to care for the patient’s needs (Year 3 & 4) Although we were in different grades and specializations, we had more fun than I thought we would, we talked as equals and had the same goals (Year 3 & 4)
6. Maturity as a nursing student is encouraged through the clinical IPE process	Reflecting on clinical IPE and making sense of its value for ourselves	There was a lot of knowledge in the textbook that actually seemed to be true because we did IPE (Year 3 & 4) There were many things that made it clear to me that this is what collaboration is all about (Year 4)
	Develop skills to deal with similar situations through clinical IPE	I have started to think about my choice of words, for example, I should explain this part to other people with additional words (Year 4).
	Putting into words what the nursing profession should be and wants to be	The IPE reaffirmed that the unique strength of nurses is to see changes in patients’ bodies and lives quickly and over time, and we need to value this (Year 4) It is the role of the nurse to inform other professionals about aspects of life and concerns (Year 4)
7. The attitudes needed for collaboration are developed	Increased interest in other professions and motivation to work on their own	I consciously looked at what the nurses and pharmacists were talking about (Year 3 & 4) We asked the pharmacists what they talked about in their drug instruction and what they paid attention to tell their patients (Year 4)
	Feel less stressed, both physically and mentally, when interacting with others who have different expertise	I don’t feel so burdened by collaboration or being involved with other people at the moment (Year 4) I can now ask people if I can join them even if they are from other professions without feeling too nervous (Year 4)

## Data Availability

The data used in this study are not open to other researchers at this time.
